# Unique Aberrations in Intimal Sarcoma Identified by Next-Generation Sequencing as Potential Therapy Targets

**DOI:** 10.3390/cancers11091283

**Published:** 2019-08-31

**Authors:** Jason Roszik, Abir Khan, Anthony P. Conley, J. Andrew Livingston, Roman Groisberg, Vinod Ravi, Roberto Carmagnani Pestana, Shiraj Sen, Vivek Subbiah

**Affiliations:** 1 Department of Genomic Medicine, Division of Cancer Medicine, The University of Texas MD Anderson Cancer Center, 1515 Holcombe Blvd., Houston, TX 77030, USA; 2 Department of Investigational Cancer Therapeutics, Division of Cancer Medicine, The University of Texas MD Anderson Cancer Center, 1515 Holcombe Blvd., Houston, TX 77030, USA; 3 Department of Sarcoma Medical Oncology, Division of Cancer Medicine, The University of Texas MD Anderson Cancer Center, 1515 Holcombe Blvd., Houston, TX 77030, USA; 4 Department of Sarcoma/Melanoma Medical Oncology, Rutgers Cancer Institute of New Jersey, 195 Little Albany St, New Brunswick, NJ 08903, USA; 5 Sarah Cannon Research Institute, Denver, CO 80218, USA

**Keywords:** intimal sarcoma, next-generation sequencing, copy number alteration, somatic mutation, gene fusion, AACR GENIE

## Abstract

Intimal sarcomas are rare and histologically heterogeneous tumors, commonly arising from the pulmonary arteries. They have remained challenging to treat. Few studies in the literature study the genomics of this cancer. Identifying targetable alterations is an important step in advancing the treatment of intimal sarcomas. Using data from the American Association for Cancer Research Project Genomics Evidence Neoplasia Information Exchange (AACR GENIE) database, we cataloged genetic alterations and assessed their clinical utility from thirteen patients with intimal sarcoma. Notable copy number alterations included amplification in *MDM2*, *CDK4*, *PDGFRA*, and *NOTCH2*, as well as copy number losses in *CDKN2A* and *CDKN2B*. Actionable alterations included mutations in *ATM/ATR*, *PTCH1*, and *PDGFRB*. Moreover, genomic rearrangement events, specifically *PDE4DIP-NOTCH2* and *MRPS30-ARID2* fusions were identified. Co-occurring alterations included a *NOTCH2* copy number gain in the *PDE4DIP-NOTCH2* fusion positive tumor and *PDGFRB* mutations in both fusion-positive cases. Our study suggests that *PDGFRB* may be relevant in the tumorigenesis process. Including genomic profiling in the management of intimal sarcoma and potential enrollment in targeted therapy trials is warranted.

## 1. Introduction

Intimal sarcomas (IS) are rare mesenchymal tumors that involve the innermost layer of large vessels, most commonly seen in pulmonary arteries [1,2,3,4]. Diagnosing this sarcoma has proved challenging despite the criteria set by the World Health Organization (WHO), as it is largely undifferentiated and histologically variable even within the same host [1,2,5,6,7]. This tumor has the ability to differentiate into other types of sarcomas such as leiomyosarcoma and angiosarcoma [1,3,5]. Since intimal sarcomas are found post-operatively or at autopsy, the true incidence of this disease entity is unknown and likely underestimated [8]. Clinical presentation can be non-specific and variable, but these tumors can mimic signs and symptoms of thromboembolism which delays diagnosis [3,5,9]. The clinical course is often aggressive, with over 50% of patients having metastasis upon diagnosis [5,10]. There are no standardized treatments or protocols for this type of cancer [11]. Intimal sarcoma has been shown to be highly resistant to conventional chemotherapies such as anthracyclines, topoisomerase inhibitors, and alkylating agents [5,11,12,13,14]. Prognosis is poor, with a median survival of 12–13 months with radical surgery aimed at complete resection [2,5,11]. The challenging dynamic on a clinical and histologic level necessitates a comprehensive genetic approach to diagnose and develop targeted treatment plans. Unfortunately, limited data is available on the molecular characteristics of this tumor.

Attempts to elucidate the genomic profile of this rare tumor have been accomplished by only a few studies, but have emphasized the involvement of *PDGFRA*, a gene encoding tyrosine kinase receptors for platelet-derived growth factor. A case report on cardiac intimal sarcoma found copy gains in *PDGFRA*, *KIT*, *STAT6*, *GLI-1*, *CKD4*, *HMGA2*, and *MDM2* (a negative regulator of p53) using whole exome sequencing (WES) and array-comparative genomic hybridization (aCGH) [4]. They also discovered concurrent *PDGFRα* amplification and *PDGFRβ* R709H mutation in intimal sarcoma [4]. The largest study of cardiac sarcomas analyzed tissue samples from 100 cases, and found intimal sarcomas to be the most frequent subtype of cardiac sarcoma, with amplification of *MDM2* in all cases. They also identified copy gains of *CDK4*, *HMGA2*, *DDIT3*, and *GLI* coinciding with 12q12–15 region via immunohistochemical analysis and confirmed with fluorescence in situ hybridization (FISH), real-time polymerase chain reaction (RT PCR), and aCGH. Other findings included copy gains in 4q12 with *KIT* and *PDGFRA*, 7p12 with *EGFR*, and copy number loss of *CDKN2A* in 9p21 [1]. Another study of IS, which was the first to propose *PDGFRA* as a molecular hallmark for intimal sarcomas, found copy number gains of *PDGFRA*, *EGFR*, and *MDM2* in 8 patients using FISH and aCGH with consistent activation of *PDGFR* and *EGFR* confirmed by western blotting [2]. Interestingly, a case report of IS in the abdominal aorta found intratumoral heterogeneity of *PDGFRA* amplification [7]. The tumor cells with the lowest degree of atypia, usually at the site of origin of the tumor, were not associated with overexpression of *PDGFRA*. Conversely, cells with the most atypia and aberrancy were found to have over-amplification of *PDGFRA*, suggesting *PDGFRA* is needed for tumorigenesis and the biopsy site is crucial for molecular studies [7]. Overall, these studies found recurrent amplification of *PDGFRA*, *PDGFRB*, *CKD4*, and *MDM2*, implicating their involvement in the tumorigenesis process. No study has found somatic mutations leading to overexpression, or unique genetic fusions. Thus, the mechanism by which these genes are overexpressed is likely to be through gene amplification [1,2,12,15].

Other malignancies with dysregulated *PDGFRA* signaling, such as gastrointestinal stromal tumors (GIST), have received treatment with tyrosine kinase inhibitors (TKI) [16]. Ex vivo assays performed on tumor cell cultures exposed to a TKI for two hours from a single patient case with IS, demonstrated a dose-response in decreasing *PDGFRA* phosphorylation confirmed by western blotting [2]. One case series had 4 of 13 patients with IS initiated on a TKI, but given the molecular complexity of the tumor, remission was not achieved [5]. Unfortunately, as these tumors involve major vessels they are excluded from any potential early phase clinical trials.

## 2. Results

**Figure 1:**
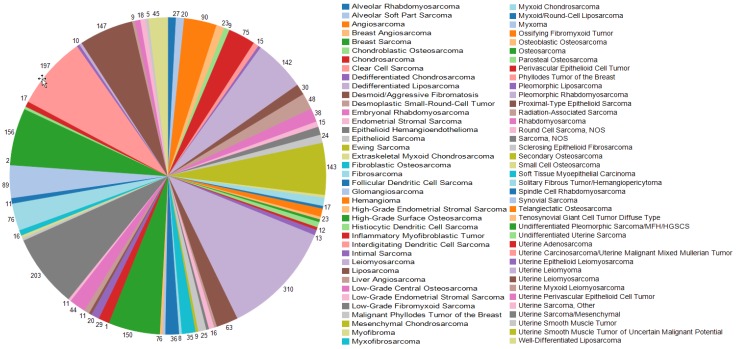
Sarcoma sample counts in American Association for Cancer Research Genomics Evidence Neoplasia Information Exchange (AACR GENIE). Color represents sarcoma subtypes as shown in the legend.

To identify common and unique genetic alterations in IS for targeted therapy, we queried data from the American Association for Cancer Research (AACR) Genomics Evidence Neoplasia Information Exchange (GENIE) genomic database and cataloged and assessed genomic alterations and their clinical utility. As shown in Figure 1, intimal sarcoma comprises only 0.6% of all sarcomas in this large database [17]. Among the 13 patients analyzed with intimal sarcoma, the median age was 46 (range 18–76); eight were female (61%) (see Table 1). Genomics were performed on tissue from the primary tumor in ten patients and metastatic site in three patients.

**Figure 2:**
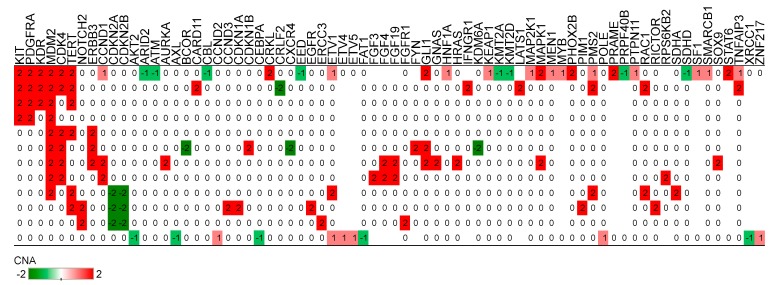
Copy number alterations in intimal sarcoma. Genes are shown in columns for patients in rows. Red color represents copy number gain while green denotes loss. The numbers show the copy number alteration (CNA) values from AACR GENIE.

Copy number alterations were available for 12 patients (see Figure 2). We identified amplifications in *MDM2* (*n* = 9, 75%), *CDK4* (*n* = 6, 50%), *TERT* (*n* = 6, 50%), *KIT* (*n* = 4, 33%), *KDR* (*n* = 3, 25%), *PDGFRA* (*n* = 4, 33%), *NOTCH2* (*n* = 2, 17%), *ERBB3* (*n* = 3, 25%), and *GLI1* (*n* = 3, 25%). Copy number losses in *CDKN2A* and *CDKN2B* were observed in three tumors (25%).

Next-generation sequencing revealed 38 somatic nonsynonymous mutations (33 missense, four frame shift, one nonsense, and one splice region) in intimal sarcoma tumors from nine patients (see Table 2). Among these mutations, M772V (c.2314A > G) and D850V (c.2549A > T) were found in *PDGFRB* from two different primary tumors. The primary tumor sample with the D850V mutation in *PDGFRB* had concurrent mutations in *PTCH1* (c.961C > A), *ATR* (c.4704C > G), and *CDKN1A* (c.419_420delGA). Potentially actionable genes are highlighted in green in Table 2.

Genomic rearrangement events included only a *PDE4DIP-NOTCH2* and a *MRPS30-ARID2* fusion (see Table 3). Interestingly, co-occurring alterations included a *NOTCH2* copy number gain in the *PDE4DIP-NOTCH2* fusion tumor and *PDGFRB* mutations in both fusion-positive cases.

We sought to analyze clinical trial results, but of the 406 sarcoma patients enrolled in clinical trials there were no patients with intimal sarcoma in a large Phase 1 clinic at the University of Texas MD Anderson Cancer Center. We hypothesize this was due to exclusion based on the location of their tumors.

## 3. Discussion

There is a large amount of heterogeneity in the presentation, histology, and genomic profile of intimal sarcomas [1,2,3,4,18]. The molecular variability and aggressive nature of this cancer makes it a challenging entity to diagnose and treat. Case reports and series have shown conventional chemotherapy with limited success, and curative radical surgery prolonging survival to a little over a year [5,11,12,13]. To our knowledge, there are no prospective clinical trials for patients with intimal sarcoma. These patients with intimal sarcoma will need to be included in clinical trials to determine which patients would benefit from genomically-matched therapies.

Our study confirmed the recurrence of copy number gains in *MDM2* and *CDK4* that coincide with the location 12q12–15, and *PDGFRA* in 4q12, as described in prior studies. The amplification of these genes strongly suggests that *PDGFR* and *MDM2* pathways are important for tumorigenesis in IS. MDM2 inhibitors under investigation have demonstrated responses in early phase clinical trials in MDM2 amplified soft tissue sarcomas (with or without CDK4 co-amplification) such as liposarcoma and synovial sarcoma [19]. Our data support the use of NGS on intimal sarcomas to identify MDM2 amplification and consideration for such trials. Thirty-three percent were found to have *PDGFRA* amplification, but this can be explained by the choice of biopsy site due to intratumoral heterogeneity [7]. New mutations in *PDGFRB* found in our study may have implications for tumorigenesis and therapeutic options as well.

We also found novel somatic mutations in another receptor kinase gene such as *ALK*, which is known to be mutated or amplified in various other cancers such as lymphomas and non-small cell lung cancers [20]. However, the ALK E1460Q mutation is outside the tyrosine kinase domain and may not be relevant clinically. Other mutations identified include ATM/ATR, and *PTCH1*, a tumor suppressor that is part of the hedgehog signaling pathway involved in tumorigenesis [21,22]. A cell checkpoint kinase mutation, which is upstream from p53 in the signaling pathway, was also seen. All of these genes could be potential targets for treatment.

Additional findings of interest include fusion proteins involving *NOTCH2-PDE4DIP* and *ARID2-MRPS30* and copy number gains in *NOTCH2*. Irregular NOTCH2 signaling has been associated with the initiation and progression of multiple cancers including liver, brain, and gastric cancers along with lymphomas [23]. In addition to NOTCH2, mutations in *ARID2*, a possible tumor suppressor, are also found in hepatocellular carcinomas [24]. The counterparts of these fusion proteins, such as MRPS30 and PDE4DIP, may also be associated with an increased risk for breast cancer and leptomeningeal disease progression, respectively [25,26]. Interestingly, both fusion proteins were found in primary tumors, but their effect on the tumorigenesis of intimal sarcomas is unknown and will require further investigation.

Limitations of this study include the sample size, which given the rarity of disease and biopsy site is likely due to intratumoral heterogeneity. Intimal sarcoma patients are currently not enrolled in clinical trials due to the location of the tumors in major vasculature. Since we noted actionable alterations in MDM2, CDK4, PDGFRA, and NOTCH2, as well as copy number losses in CDKN2A and CDKN2B, our study has clinical implications. There are now three US FDA approved CDK4/6 inhibitors for breast cancer, Palbociclib (IBRANCE^®^, Pfizer Inc.), ribociclib (Kisqali^®^, Novartis Pharmaceuticals Corporation), and abemaciclib (VERZENIO^™^, Eli Lilly and Company). Given the multiple aberrations seen in the CDK pathway, CDK4/6 inhibitors may be an option in clinical trials or off-label based on molecular profiling data. In addition, some of these tumors may be responsive to drugs that are already approved for the treatment of sarcomas like Pazopanib (Votrient^®^), which is a multi-kinase VEGF based inhibitor that has activity against the PDGFR pathway as well. Other novel TKIs and MDM2 antagonists should be evaluated as treatments for advanced IS. In addition, the role of ATM/ATR, NOTCH2, and PTCH1 should be studied as potential therapeutic targets. Investigators should be encouraged to enroll these patients with specific molecular aberrations to basket trials.

## 4. Materials and Methods

We queried the AACR Project Genomics Evidence Neoplasia Information Exchange (GENIE) database (version 5) [27]. The GENIE registry derives existing CLIA-/ISO-certified genomic data [28]. Published records in Pubmed/Medline were reviewed for genomic data from several different platforms. Retrospective records were abstracted to appraise the benefit of using a targeted therapy approach in a large Phase 1 clinic at the University of Texas MD Anderson Cancer Center.

Ethics Statement: All AACR GENIE project data has been de-identified using the HIPAA Safe Harbor Method. Analyses were retrospective and were performed in accordance with the AACR GENIE Human Subjects Protection and Privacy policy. Institutional Review Board (IRB) details are provided in the AACR GENIE Data Guide [28].

## 5. Conclusions

Comprehensive genomic profiling may be considered for intimal sarcoma, both to confirm diagnosis and to explore treatment options on clinical trials. Clinical trial enrollment should be considered for all intimal sarcomas, and further exploration of copy number changes and genetic alterations is needed for this relatively chemotherapy-resistant disease. A global multicenter prospective registry/trial would best determine the response of this rare cancer to targeted therapies.

## Figures and Tables

**Table 1:** Patient characteristics of the AACR GENIE intimal sarcoma patients.

Patient	Cancer Type Detailed	Sample Type Detailed	Age at Seq Report	Sex	Primary Race	Ethnicity
Patient 1	Intimal Sarcoma	Metastasis site unspecified	26	Male	Asian	Non-Spanish/non-Hispanic
Patient 2	Intimal Sarcoma	Primary tumor	20	Male	White	Non-Spanish/non-Hispanic
Patient 3	Intimal Sarcoma	Metastasis site unspecified	42	Male	White	Non-Spanish/non-Hispanic
Patient 4	Intimal Sarcoma	Primary tumor	47	Female	White	Non-Spanish/non-Hispanic
Patient 5	Intimal Sarcoma	Primary tumor	46	Female	Asian	Non-Spanish/non-Hispanic
Patient 6	Intimal Sarcoma	Primary tumor	18	Female	White	Spanish/Hispanic
Patient 7	Intimal Sarcoma	Primary tumor	34	Male	White	Non-Spanish/non-Hispanic
Patient 8	Intimal Sarcoma	Primary tumor	69	Female	White	Non-Spanish/non-Hispanic
Patient 9	Intimal Sarcoma	Metastasis site unspecified	76	Female	White	Non-Spanish/non-Hispanic
Patient 10	Intimal Sarcoma	Primary tumor	56	Female	White	Non-Spanish/non-Hispanic
Patient 11	Intimal Sarcoma	Primary tumor	46	Male	White	Non-Spanish/non-Hispanic
Patient 12	Intimal Sarcoma	Primary tumor	71	Female	White	Non-Spanish/non-Hispanic
Patient 13	Intimal Sarcoma	Primary tumor	49	Female	Asian	Unknown

**Table 2:** Somatic mutations in intimal sarcoma samples. Potentially actionable genes are highlighted in green. (Potentially actionable genes are highlighted in green.)

Sample	Gene	Variant Classification	Mutation
Patient 1	ASXL1	Missense	D864E
Patient 1	ASXL1	Missense	G1299R
Patient 1	GLI1	Missense	R171Q
Patient 1	IDH2	Missense	I153V
Patient 1	KDR	Splice Region	
Patient 1	MDM2	Intron	
Patient 1	MYD88	Silent	D247D
Patient 1	NF1	Missense	L121V
Patient 1	RARA	Intron	
Patient 1	RET	Missense	M1009T
Patient 1	SETBP1	Missense	P1526Q
Patient 2	ARHGEF12	Missense	E186K
Patient 2	KDM5A	Missense	S1408N
Patient 2	TMEM127	Missense	I188V
Patient 2	TOPBP1	Missense	L1499P
Patient 3	ERBB4	Missense	H893R
Patient 5	BARD1	Missense	A502N
Patient 5	ERCC5	Missense	S453C
Patient 5	SOX2	Missense	A263E
Patient 5	TP53	Missense	R273C
Patient 5	U2AF1	Missense	G212A
Patient 7	ATR	Missense	D1568E
Patient 7	CDKN1A	Frame Shift Del	R140Qfs*56
Patient 7	FAT1	Missense	P1333L
Patient 7	PDGFRB	Missense	D850V
Patient 7	PTCH1	Missense	L321I
Patient 7	RECQL4	Missense	E37K
Patient 8	ALK	Missense	E1460Q
Patient 8	GATA1	Missense	G165S
Patient 8	GRIN2A	Missense	R1309Q
Patient 8	INPP4A	Missense	P773S
Patient 8	JAK3	Missense	W716R
Patient 8	NOTCH4	Missense	A1439T
Patient 8	PAK7	Frame Shift Del	P612Lfs*3
Patient 10	PDGFRB	Missense	M772V
Patient 11	EIF1AX	Missense	G6R
Patient 11	ERCC2	Missense	T49A
Patient 11	MDM4	Missense	D173H
Patient 11	NSD1	Nonsense	Q2274*
Patient 11	SETD2	Frame Shift Del	V1070Lfs*44
Patient 12	EP300	Missense	S2404A
Patient 12	HGF	Frame Shift Ins	T4Gfs*39
Patient 13	ATM	Intron	

**Table 3:** Gene fusions in intimal sarcoma. Comments are from the GENIE database.

Sample	Fusion	DNA Support	RNA Support	Frame	Comments
Patient 7	PDE4DIP-NOTCH2	yes	unknown	in frame	Note: The PDE4DIP (NM_022359)-NOTCH2 (NM_024408) rearrangement event is a deletion which results in the fusion of PDE4DIP exon 1 and NOTCH2 exons 27–34. Its functional significance is undetermined.
Patient 10	MRPS30-ARID2	yes	unknown	unknown	ARID2 (NM_152641) rearrangement: t(5;12) (p12;q12) (chr5:g.44601610::chr12:g.46245800) Note: The ARID2 rearrangement is a translocation which results in the truncation of ARID2 exons 15–21. One of the breakpoints is within ARID2 exon15.
